# 8-OxodG: A Potential Biomarker for Chronic Oxidative Stress Induced by High-LET Radiation

**DOI:** 10.3390/dna4030015

**Published:** 2024-08-01

**Authors:** Kamendra Kumar, Albert J. Fornace, Shubhankar Suman

**Affiliations:** 1 Department of Oncology, Lombardi Comprehensive Cancer Center, Georgetown University Medical Center, Washington, DC 20057, USA; 2 Department of Biochemistry and Molecular & Cellular Biology, Georgetown University Medical Center, Washington, DC 20057, USA

**Keywords:** oxidative DNA damage, high-LET radiation, space radiation

## Abstract

Oxidative stress-mediated biomolecular damage is a characteristic feature of ionizing radiation (IR) injury, leading to genomic instability and chronic health implications. Specifically, a dose- and linear energy transfer (LET)-dependent persistent increase in oxidative DNA damage has been reported in many tissues and biofluids months after IR exposure. Contrary to low-LET photon radiation, high-LET IR exposure is known to cause significantly higher accumulations of DNA damage, even at sublethal doses, compared to low-LET IR. High-LET IR is prevalent in the deep space environment (i.e., beyond Earth’s magnetosphere), and its exposure could potentially impair astronauts’ health. Therefore, the development of biomarkers to assess and monitor the levels of oxidative DNA damage can aid in the early detection of health risks and would also allow timely intervention. Among the recognized biomarkers of oxidative DNA damage, 8-oxo-7,8-dihydro-2′-deoxyguanosine (8-OxodG) has emerged as a promising candidate, indicative of chronic oxidative stress. It has been reported to exhibit differing levels following equivalent doses of low- and high-LET IR. This review discusses 8-OxodG as a potential biomarker of high-LET radiation-induced chronic stress, with special emphasis on its potential sources, formation, repair mechanisms, and detection methods. Furthermore, this review addresses the pathobiological implications of high-LET IR exposure and its association with 8-OxodG. Understanding the association between high-LET IR exposure-induced chronic oxidative stress, systemic levels of 8-OxodG, and their potential health risks can provide a framework for developing a comprehensive health monitoring biomarker system to safeguard the well-being of astronauts during space missions and optimize long-term health outcomes.

## 1. Background

**Figure 1.**
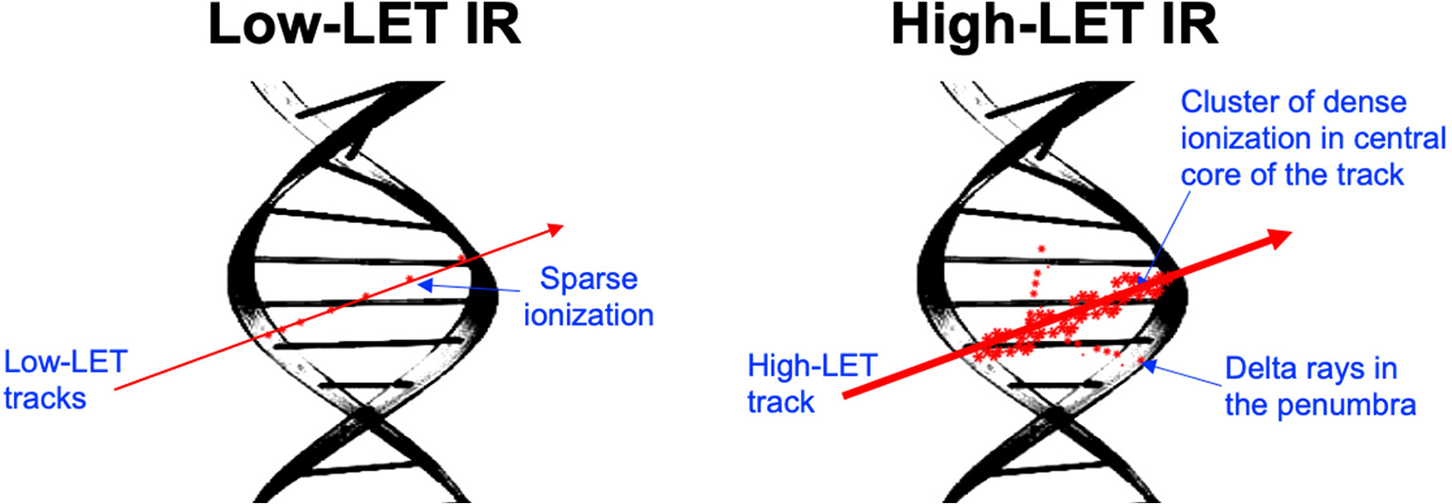
Illustration of a simplified track structure depicting energy deposition (ionization) after low- and high-LET (linear energy transfer) IR (ionizing radiation) exposure.

Ionizing radiation (IR) possesses sufficient energy to cause the ionization of biomolecules and water (H_2_O) present within the tissue or organ in the field of irradiation [1,2]. Based on ionization characteristics, IR is broadly classified as either low- and high-linear energy transfer (LET) radiation. Photons (X-rays or γ-rays) are considered low-LET IR, as they distribute energy uniformly, causing sparse ionization across the tissue volume. Contrary to photons, high-LET IR, including proton (hydrogen nucleus or H^+^), alpha particle (helium nucleus), and heavy ion (atomic number Z > 2, such as Carbon, Oxygen, Silicon, Iron, etc.) radiation, decelerates rapidly due to their inherent mass, resulting in an escalating energy deposition as they penetrate through the target tissue [2,3]. The rate of energy loss depends on the particle type, energy, and the density of the target material. Initially, the LET may increase with depth, reaching a maximum (Bragg peak), and then rapidly decrease as the particle loses all its energy. The Bragg peak represents the point where the LET is highest, occurring just before the particle comes to a stop [4,5]. Energy deposition via high-LET IR follows a track pattern (Figure 1), characterized by a central region of extremely high energy density with lateral dimensions confined to the submicroscopic scale, encircled by a penumbra consisting of sporadically ionized electrons or δ-rays [4,5]. The nuclear interactions in the central core region can also generate neutrons, δ-rays, and secondary charged particles with varying mixed LET characteristics [4,6,7], resulting in highly heterogeneous energy deposition at the submicroscopic scale. Therefore, accurate dosimetry characterization and the modeling of the spatial energy distribution are crucial for understanding and simulating the energy deposition patterns and the subsequent DNA damage following high-LET IR exposure [8,9].

Both low- and high-LET IR exposure can impact DNA through direct or indirect mechanisms, but the degree of these effects depends on factors such as energy, charge, mass, and the cell types in the field of irradiation. Generally, the direct effect of IR involves the generation of ion pairs and free radicals within the DNA, leading to the creation of cation and anion radicals [4]. Additionally, a quasi-direct effect where the ionization of the initial few water molecules surrounding the DNA molecule leads to the efficient transfer of holes and electrons to the DNA, resulting in the formation of more cation and anion radicals on the DNA observed after high-LET exposure [10]. Physico-chemical studies have also corroborated that the production of DNA radicals corresponds to the increase in the LET of the ion beam along the beam track [11]. Contrary to the direct effect, indirect effects occur when surrounding water molecules undergo ionization, leading to the formation of free radicals, which further results in oxidative DNA damage. The time scale of the indirect and direct effects of IR can vary significantly depending on the dose, dose rate, and LET, as well as the exposed biological system [12,13]. Generally, direct effects of IR manifest almost instantly after exposure and are typically observed within microseconds to milliseconds and depending on the severity of the damage caused by direct radiation interactions; cellular responses such as repair mechanisms or cell death can occur within minutes to hours following exposure. Indirect effects of IR occur when radiation interacts with water molecules in the cells, producing reactive oxygen species (ROS) and other free radicals. These reactive species can then go on to damage cellular components, including DNA. The onset of indirect effects can be delayed compared to direct effects and may occur within minutes to hours after exposure [4,14]. Very short-lived ROS with unpaired electrons are classified as free radicals, including, superoxide anions (O2•^−^), hydroxyl radicals (HO•), nitric oxide radicals (•NO), and lipid radicals. Additionally, other molecules with oxidizing capabilities, include, hydrogen peroxide (H_2_O_2_), peroxynitrite (ONOO^−^), and hypochlorous acid (HOCl) [15–17]. Indirect ROS production also involves the activation of cellular metabolic processes, including mitochondrial respiration and enzymatic reactions [18–22]. Therefore, it is widely acknowledged that exposure to IR can induce oxidative stress, a condition characterized by an imbalance between ROS production and the antioxidant defense system, leading to cellular damage, including to DNA, proteins, and lipids, ultimately contributing to various pathophysiological processes [23].

Among the myriad biomolecular target’s nucleic acids (i.e., DNA and RNA) are particularly susceptible to oxidative damage, and if the damage is not adequately repaired, it can lead to mutations or cellular dysfunction, which may manifest as long-term adverse health consequences [23–25]. Exposure to IR can induce a cascade of DNA lesions, including single-strand breaks (SSBs), double-strand breaks (DSBs), and oxidative base modifications. High-LET IR has been demonstrated to induce clustered DNA damage marked by a multiply damaged site (MDS) within a short segment of DNA, such as DSBs, along with base damage and SSBs in close proximity [26–28]. This complexity increases the difficulty in DNA repair and enhances the likelihood of errors during the repair process. Notably, the interplay between clustered DNA damage and chronic oxidative stress is particularly pronounced following high-LET radiation exposure, as high-LET IR induces significant ROS production, which, when coupled with the inherently severe DNA lesions, amplifies its biological impact. The resulting oxidative environment further enhances the clustering of DNA damage, impedes effective repair, and perpetuates a cycle of damage and cellular dysfunction [29,30]. The sustained presence of ROS not only exacerbates initial damage but also promotes secondary lesions, contributing to the complexity and persistence of DNA damage. Additionally, irradiated cells with persistent DNA damage signals are likely to undergo permanent cell cycle arrest (senescence), or cell death if the damage is irreparable. Senescence is considered a barrier to the proliferation of damaged cells and has been implicated in preventing oncogenic transformation after IR exposure. However, senescent cells are metabolically inactive, and they can adopt a complex secretory phenotype known as the Senescence-Associated Secretory Phenotype (SASP), which involves the secretion of various pro-inflammatory cytokines, chemokines, growth factors, and proteases. These factors are known to further exacerbate oxidative stress, leading to a vicious cycle of sustained DNA damage and further senescence/SASP induction [14,19,21,25].

Notably, chronic oxidative stress-associated DNA damage persists long and displays multifold higher levels of biomolecular oxidative damage after high-LET radiation exposure, compared to the same dose of photon radiation [3,21,25]. One of the most prevalent forms of oxidative DNA damage is the formation of 8-oxo-7,8-dihydro-2′-deoxyguanosine (8-OxodG), a modified nucleoside resulting from the oxidation of guanine residues in DNA [25,31]. The accumulation of 8-OxodG in cellular DNA is considered a hallmark of chronic oxidative stress and has been implicated in diseases, including inflammation, increased risk of cancer, and neurodegenerative and cardiovascular diseases, that can develop over months to years following IR exposure [32–34]. Accumulating evidence suggests that the measurement of 8-OxodG levels in biological samples, including blood, urine, and tissues, serves as a biomarker for assessing the extent of oxidative DNA damage and the associated health risks following IR exposure [35]. Elevated levels of 8-OxodG have been observed in individuals exposed to various sources of IR, including medical diagnostic procedures, occupational settings, and environmental contaminants [36]. This review explores the potential of 8-OxodG as a biomarker for chronic stress induced by high-LET radiation, focusing on its sources, formation, repair, and detection methods. By understanding the connection between high-LET IR-induced 8-OxodG and its potential health implications, we can establish this as a biomarker to monitor astronaut’s health during long-duration space missions and ensure their well-being.

## 2. Human Exposure to High-LET Radiation

Contrary to widespread low-LET radiation exposure through diagnostic radiology [37], cancer radiotherapy [38], and occupational exposures [39], the scenarios of high-LET radiation exposure are quite limited: (1) astronauts undertaking deep space exploration beyond Earth’s magnetosphere [40,41], (2) patients undergoing high-LET cancer radiotherapy using proton or carbon beams [42], (3) exposure to neutron radiation originated from a radiological dispersal device (RDD), dirty bomb, or nuclear disaster involving uncontrolled nuclear fission chain reactions or criticality excursion [43,44], and (4) radon miners [45]. Animal model studies have suggested that the biological effects of high-LET IR depend on factors such as the dose, LET, and sex [19,20,25,46–51]. In particular, deep space missions, such as those to Mars or beyond, involve prolonged exposure to cosmic radiation, which includes high-LET radiation with Z > 2 from galactic cosmic rays (GCR) that can penetrate through spacecraft shielding [40,41,52,53]. Moreover, prolonged exposure to high-LET radiation can increase the risk of cancer, cataracts, central nervous system effects, and other health issues [3,54–56]. Unlike low-LET radiation, the molecular mechanism and associated biomarkers used to assess long-term health impacts of high-LET space radiation are not fully understood, and are being studied using mouse models irradiated to simulated space radiation on Earth [57,58]. Having a biomarker specific to high-LET radiation exposure would enable the early detection of any potential health effects. By monitoring these biomarkers, medical professionals could intervene earlier to mitigate health risks, possibly by adjusting mission durations, shielding strategies, or medical treatments.

## 3. Potential Sources of 8-OxodG

**Figure 2.**
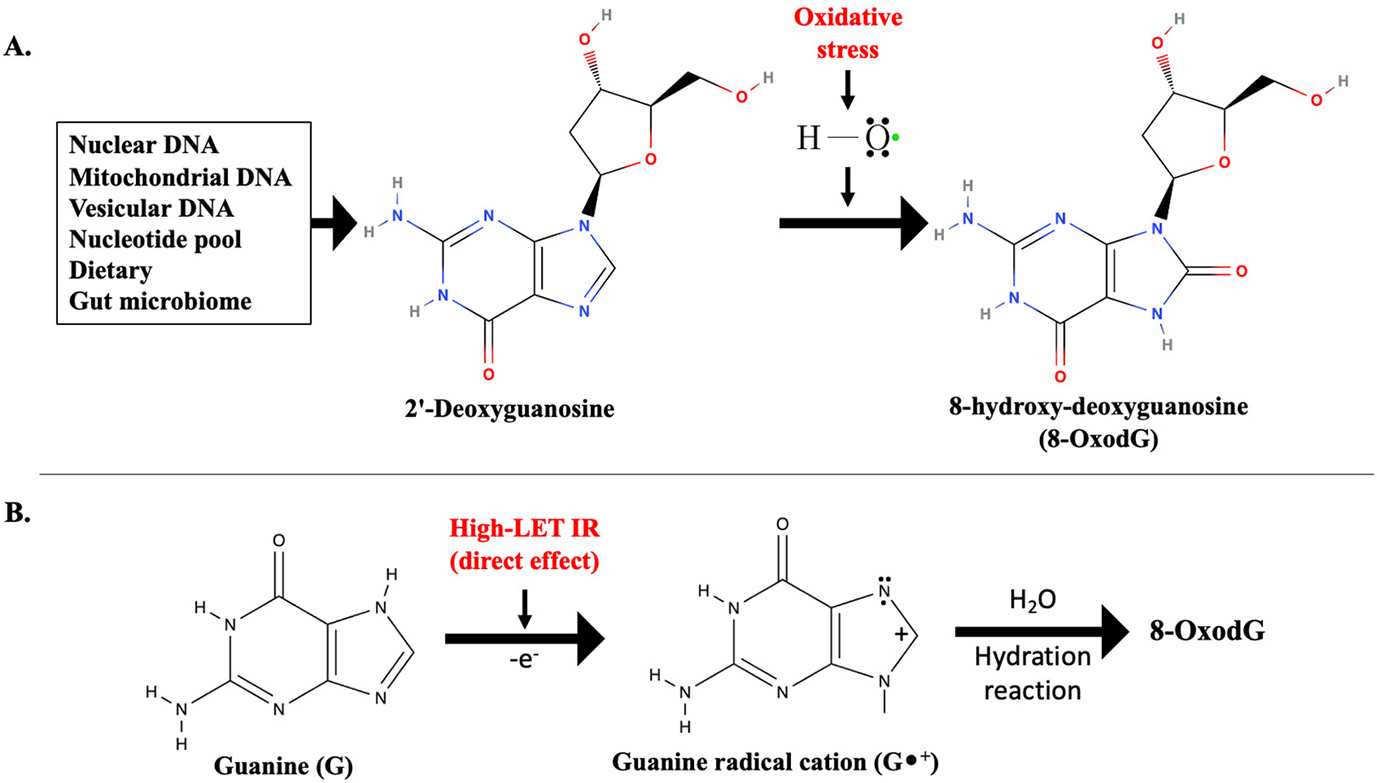
Potential cellular and non-cellular sources of 2′-deoxyguanosine and formation of 8-OxodG. (**A**) Oxidative stress-induced 8-OxodG formation in presence of hydroxyl radical. (**B**) 8-OxodG formation through direct effect of high-LET IR, involving one-electron oxidation followed by hydration reaction.

Guanine (G) is present in both DNA and RNA and is considered highly susceptible to oxidation due to the presence of its reactive electron-rich structure [59,60]. The production of 8-OxodG in DNA and 8-OxoG (8-oxo-7,8-dihydroguanosine) in RNA involves complex chemical reactions where hydroxyl radicals generated by IR can abstract a hydrogen atom from the C8 position of guanine, leading to the formation of a guanine radical (G•). The G• is highly reactive, and the addition of oxygen to the G• radical results in the formation of a guanine peroxyl radical (G-OO•). This intermediate can further react with molecular oxygen to form 8-OxodG (Figure 2A). Additionally, due to low redox potential, guanine is highly susceptible to undergo one-electron oxidation, which results in the formation of a guanine radical cation (G•^+^) [61]. In duplex DNA, G•^+^ has been demonstrated to undergo hydration to form 8-OxodG [62]. Notably, high-LET IR is more likely to induce direct ionization, resulting in the formation of G•^+^ compared to low-LET radiation [63,64] (Figure 2B).

The formation chemistry of 8-OxodG and 8-OxoG, respectively, in DNA and RNA shares similarities, and some notable differences; for example, the presence of the 2′-OH group in RNA can potentially increase its susceptibility to oxidative damage compared to DNA [65]. Both 8-OxodG and 8-OxoG are considered biomarkers of oxidative stress [35]; however, 8-OxodG is considered a better biomarker of oxidative stress than 8-OxoG because of its higher systemic stability [66,67]. This means that 8-OxoG levels may not accurately reflect the extent of oxidative DNA damage, whereas 8-OxodG levels provide a more stable and reliable measure [35]. Moreover, 8-OxodG is specific to DNA, whereas 8-oxoG largely comes from RNA or the cellular nucleotide pool [66,67]. This specificity makes 8-OxodG a more precise biomarker for assessing oxidative damage to DNA, which is particularly relevant for studying diseases associated with DNA damage. Increased systemic levels of 8-OxodG have been demonstrated to be linked to increased oxidative stress after whole-body IR exposure [68]. The potential sources of 8-OxodG known to contribute to the systemic levels of 8-OxodG under normal and stressed conditions are described below.

### 3.1. Cellular Sources of 8-OxodG

**Figure 3.**
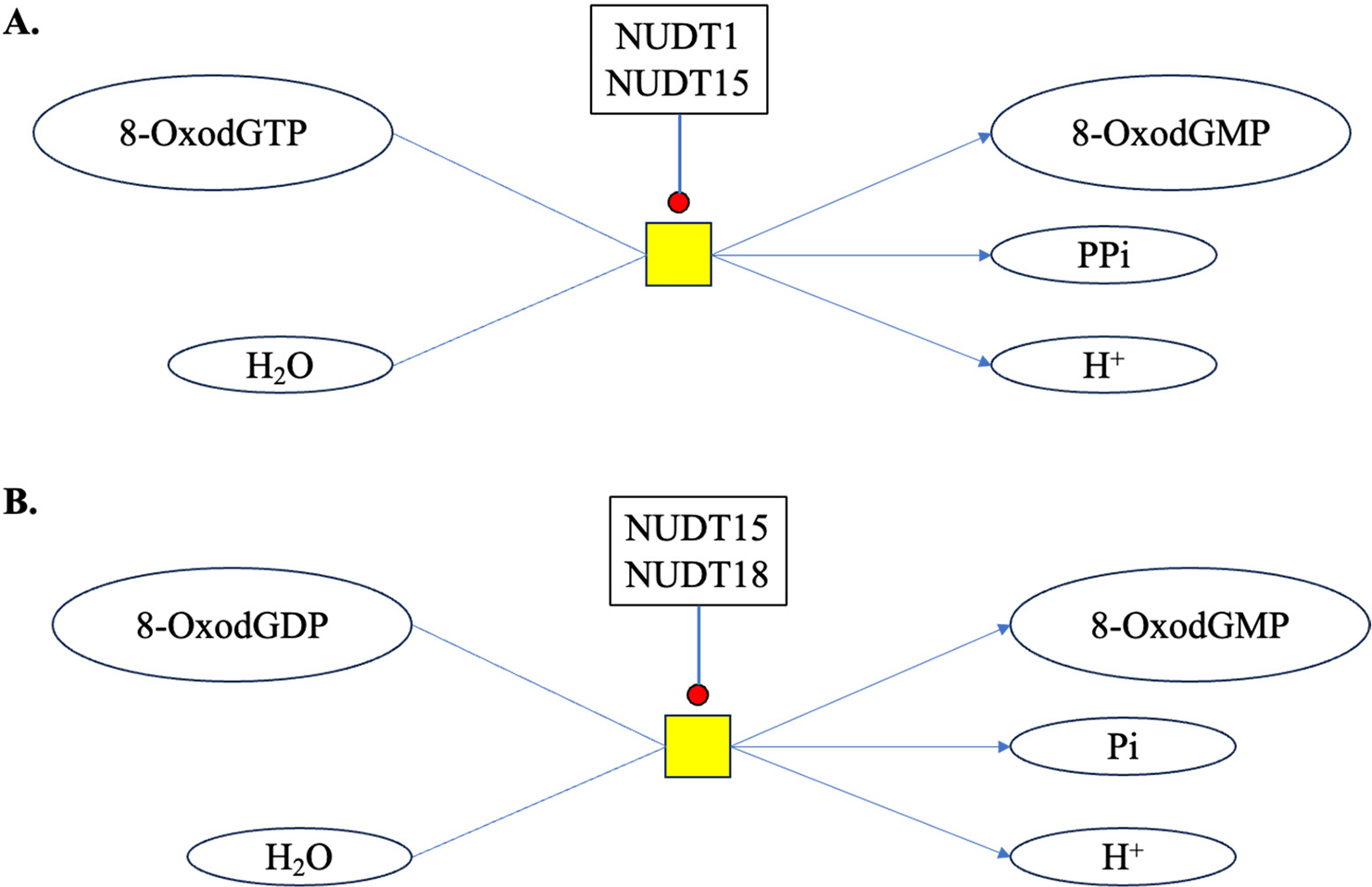
Catalysis of 8-OxodG by Nudix superfamily enzymes in cytosol. (**A**) NUDT1 (MTH1, MutT homolog 1) and NUDT15 (MTH2, MutT homolog 2) catalyzes the reaction of 8-OxodGTP and water to form 8-OxodGMP and PPi (pyrophosphate). (**B**) NUDT15 (MTH2) and NUDT18 (MTH3, MutT homolog 3) catalyzes the reaction of 8-OxodGDP and water to form 8-OxodGMP and Pi (orthophosphate).

Cells have also evolved various mechanisms to counteract oxidative damage to the nuclear (genomic) DNA, including antioxidant defense systems and DNA repair pathways [69]. During repair, enzymes like 8-Oxoguanine DNA glycosylase (OGG1) remove the damaged 2^′^-deoxyguanosine, leaving behind an apurinic/apyrimidinic (AP) site and releasing 8-OxodG into the extracellular space [70,71]. In addition to genomic DNA, mitochondria also possess their own DNA, i.e., mtDNA, which is more susceptible to oxidative damage relative to its nuclear counterpart because of its close proximity to ROS-generating processes, increasing the likelihood of oxidative damage [72]. The mtDNA lacks histones and has less robust DNA repair mechanisms, making it more susceptible to damage from ROS [73]. Multifold increases in 8-OxodG have been reported in the mtDNA compared to nuclear DNA after high-LET IR exposure, relative to low-LET IR [74]. Moreover, IR-induced oxidative stress can lead to the accumulation of mtDNA damage, resulting in mitochondrial dysfunction, further driving a vicious cycle of ROS production resulting in long-term persistence of oxidative stress [75,76]. In addition to 2^′^-deoxyguanosine present in DNA, a cell also maintains a pool of nucleotides for nucleic acid synthesis [77]. Damaged nucleotides like 8-OxodGTP can be hydrolyzed by enzymes like NUDT (Nudix) hydrolases. NUDT1 or MTH1 (MutT homolog 1) catalyzes the reaction of 8-OxodGTP and water to form 8-OxodGMP and PPi (pyrophosphate). Nudix superfamily enzymes (i.e., NUDT1, NUDT15 and NUDT18) are known to catalyze the hydrolysis of phosphodiester bonds in molecules including nucleoside triphosphates and diphosphates and nucleotide sugars [78] (Figure 3). The hydrolysis of 8-OxodGTP and 8-OxodGDP may protect the cell from incorporating these damaged nucleosides into the DNA [79,80], but can also release 8-OxodG into the extracellular space and finally into urine.

### 3.2. DNA Trapped in Vesicles

In case of lethally damaged cells through processes like apoptosis or necrosis, their contents, including damaged DNA fragments containing 8-OxodG, can leak into the extracellular space. Additionally, intracellular vesicles (IVs) and extracellular vesicles (EVs), including exosomes, microvesicles, and apoptotic bodies, are membrane-bound vesicles secreted by cells and play crucial roles in intercellular communication and the transfer of biomolecules between cells [81,82]. The oxidative damage to DNA within these vesicles is also known to occur through endogenous ROS generation as well as external sources of oxidative stress, such as inflammatory mediators, which can also induce oxidative damage to DNA present within the vesicles [83,84]. Oxidative damage to DNA within vesicles has been implicated in various disease conditions, including cancer, neurodegenerative disorders, and cardiovascular diseases [85]. Furthermore, the transfer of oxidatively damaged DNA is also known to cause oxidative stress in the recipient cells [86,87], potentially leading to the release of more 8-OxodG into the extracellular space fueling a vicious cycle of persistent oxidative stress and the chronic production of 8-OxodG.

### 3.3. Dietary and Microbiome Released 8-OxodG

In addition to 8-OxodG generated within tissue and vesicles, exogenous sources, such as diet and microbiome, can also contribute to systemic levels of 8-OxodG. 8-OxodG has been detected in many meat-based food products [88,89], and the intake of processed foods could also contribute to systemic levels of 8-OxodG [90,91]. Additionally, studies have also suggested a potential link between the gut microbiome and levels of 8-OxodG. Interestingly, the levels of 8-OxodG in the small intestine of germ-free mice were significantly lower than those in the conventionally raised group [92]. The gut microbiome can also produce or modulate ROS levels, and can therefore affect the body’s ability to neutralize ROS and repair damaged DNA, further influencing the 8-OxodG levels [93]. Both low- and high-LET IR exposure are known to alter the gut microbiome, which in turn influence the systemic levels of 8-OxodG [94–96].

## 4. High-LET Radiation-Induced Chronic Oxidative Stress

To meet the demands of cellular physiology, small amounts of ROS are normally produced within a cell [97]. In mitochondria, ATP is produced through the electron transport chain (ETC), and as small amount of superoxide radical is also produced. Further, the mitochondria-specific superoxide dismutase-2 (SOD2) enzyme catalyzes the transformation of superoxide radicals to hydrogen peroxide, which is later converted to water and oxygen by catalase [98]. Fan et al. reported that the mitochondrial ETC is significantly activated after high-LET IR exposure, resulting in the overproduction of superoxide and hydrogen peroxide due to mitochondrial dysfunction [99]. Besides mitochondria, the endoplasmic reticulum (ER) can also generate ROS during the unfolded protein response (UPR) through the activation of ER oxidoreductases, leading to the production of hydrogen peroxide [100]. Other enzymatic sources of ROS also include, xanthine oxidase (XO), NADPH oxidases (NOX), uncoupled NO synthase, cytochrome p450, peroxidases, and cyclooxygenases [15].

Cellular oxidative stress is one of the known hallmarks of IR exposure [101–103]. Moreover, oxidative stress is commonly implicated in wide-ranging IR-related health effects, including inflammation, accelerated aging, and carcinogenesis [104–106]. Several studies suggest that IR can trigger both acute and long-lasting oxidative stress and subsequent increases in oxidative DNA damage [20,21,47,101,107]. Comparative studies using low- and high-LET IR-exposed in vitro and in vivo model systems have been conducted to understand both the acute and delayed onset of oxidative stress involving ROS generation through mitochondria and other enzymes, antioxidant system dysfunction, and accumulations of oxidatively damaged biomolecules [17,19,20,25,47,108]. Moreover, increasing evidence suggests that high-LET IR exposure is highly potent in inducing long-lasting oxidative stress relative to low-LET IR [20,47,108]. For example, significant increases in both acute and persistent oxidative stress in monolayer culture of neural cells have been noted after high-LET IR, compared to photon exposure. A study using neurospheres and brain organoids grown from multipotent neural cells have also displayed higher oxidative stress after high-LET IR exposure [109,110]. Additionally, in vivo analyses of persistent oxidative stress and 8-OxodG production in mouse brain months after IR-exposure have also indicated a greater potential of high-LET IR to cause persistent oxidative stress and subsequent increases in 8-OxodG [20]. Skin fibroblasts exposed to X-rays and ^12^C-ions have also suggested radiation quality to be dependent on greater oxidative damage and the downregulation of antioxidant defense systems up to three weeks post irradiation [111]. High-LET irradiation also alters redox-related genes during osteoblastogenesis, which may contribute to persistent bone loss [112]. Mechanisms involved in persistent oxidative stress after high-LET IR includes relatively higher mitochondrial superoxide production, mitochondrial dysfunction, increased NADPH oxidase, and cyclooxygenase-2 activity alongside decreased antioxidant defenses, compared to low-LET IR exposure [20,21,47,99,113].

**Figure 4.**
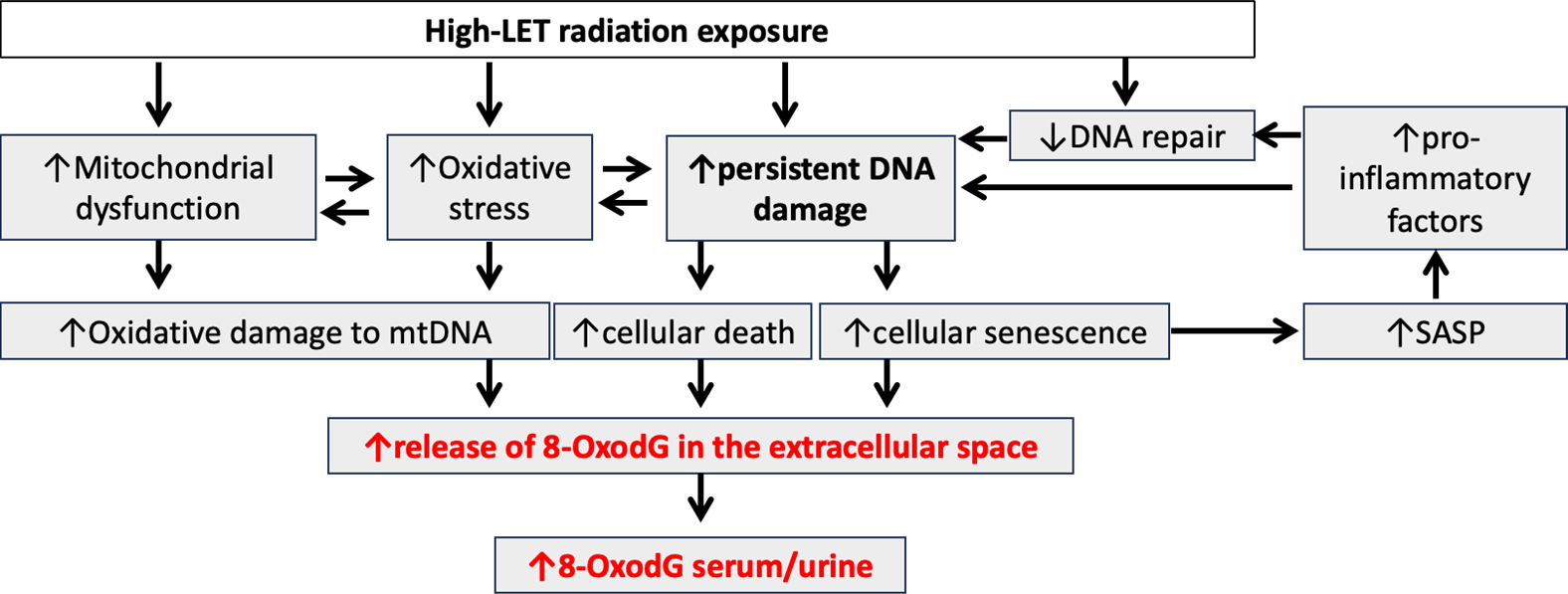
Illustration of potential mechanisms involved in sustained increases in serum and urine levels of 8-OxodG after high-LET ionizing radiation exposure. High-LET radiation exposure causes a cascade of detrimental effects, including mitochondrial dysfunction, increased ROS generation, and oxidative damage to mitochondrial DNA, which further compromises mitochondrial function. Elevated ROS levels damage cellular components, including nuclear DNA. Persistent accumulation of unrepaired DNA lesions and impaired DNA repair exacerbate DNA damage, leading to cell death and senescence. Senescent cells can adopt a Senescence-Associated Secretory Phenotype (SASP), secreting pro-inflammatory factors and proteases that perpetuate DNA damage and induce further senescence/SASP, creating a vicious cycle. Arrows: ↑ (increase) and ↓ (decrease).

High-LET radiation is generally more efficient in inducing chronic 8-OxodG formation per unit dose compared to low-LET radiation [25]. Prevost et al. reported 1.5-fold higher levels of extracellular 8-OxodG at 1 and 14 days post ^12^C-ion exposure in normal human skin fibroblasts compared to an equivalent dose of X-rays [114]. Irrespective of IR-types, the kinetics of 8-OxodG formation typically follow a dose-dependent pattern, with increasing dose up to the lethality threshold levels of 8-OxodG is expected to increase, but as repair mechanisms come into play, the levels of 8-OxodG could decrease over time, reflecting the repair of damaged DNA. Therefore, the persistence of 8-OxodG in cells exposed to sublethal doses of IR is likely to be influenced by the balance between its formation and repair mechanisms [115,116]. Factors such as the cell type, the presence of antioxidants, and the efficiency of DNA repair pathways can influence the time kinetics of 8-OxodG formation and persistence [117]. Base Excision Repair (BER) plays a significant role in rectifying DNA base damage caused by oxidation, while Nucleotide Excision Repair (NER) is involved to a lesser extent in repairing such lesions [25,118]. BER is a DNA repair mechanism correcting small base lesions, facilitated by DNA glycosylases removing damaged bases. The remaining apurinic/apyrimidinic (AP) site undergoes endonuclease activity, forming a single-strand break (SSB). This gap is filled and rejoined by replacing the AP site with a single-nucleotide match (short-patch BER) or synthesizing a few nucleotides (long-patch BER). BER uses various glycosylases for different types of damage induced by oxidation, alkylation, and deamination. OGG1 targets 8-OxodG, generating an AP site, further processed by AP endodeoxyribonuclease 1 (APE1) and Poly(ADP-Ribose) Polymerase 1 (PARP1), and Poly(ADP-Ribose) Polymerase 1 (PARP2) occupy SSBs, recruiting downstream repair proteins. DNA polymerase beta (Pol β) fills and ligates SSBs, undergoing short- or long-patch BER [25,31]. Additional repair pathways involve MutY DNA Glycosylase (MUTYH), Nei Like DNA Glycosylase 1(NEIL1), MTH1, transcription coupled-NER (TC-NER), and mismatch repair (MMR). These pathways overlap, serving as backups for OGG1-mediated BER [119]. In a nutshell, persistent elevated levels of ROS, mitochondrial dysregulation, and the downregulation of both BER- and NER-related gene and protein expressions reported earlier [21,25,47] are key mechanisms for the long-term persistence of 8-OxodG and subsequent increase in systemic levels after exposure to high-LET heavy ions (Figure 4).

## 5. 8-OxodG as a Biomarker of High-LET Radiation-Induced Chronic Oxidative Stress and Associated Adverse Health Effects

**Figure 5.**
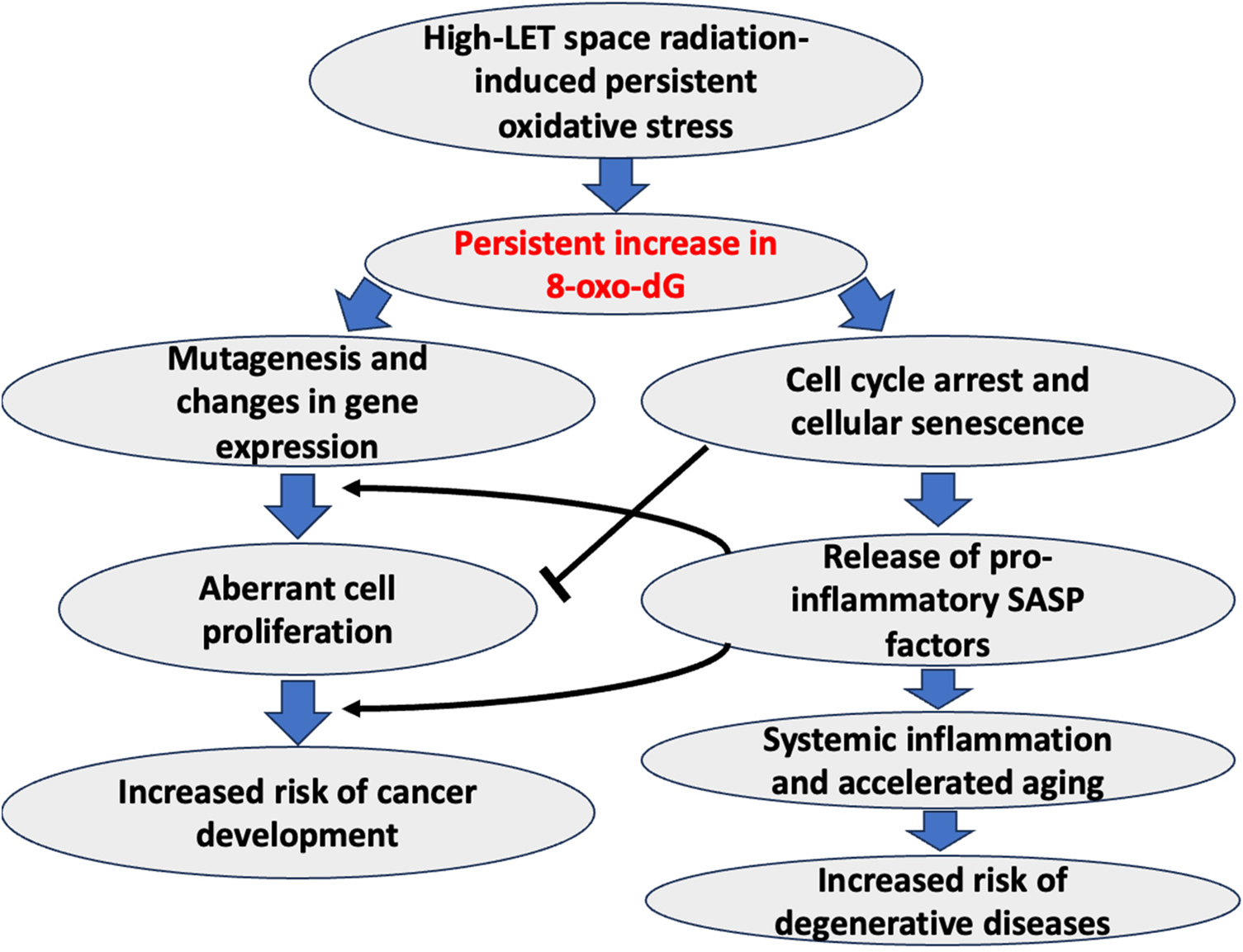
A schematic of the potential involvement of high-LET space radiation-induced persistent 8-OxodG formation and higher-risk cancer and degenerative diseases. The pointed black arrow indicates activation, whereas the blunt arrow indicates an inhibitory effect.

The accumulation of oxidative damage to DNA can also trigger premature aging processes, leading to the deterioration of various physiological functions and an overall decline in health. Expectantly, high-LET space radiation-induced persistent oxidative stress poses a myriad of health risks, including inflammation, carcinogenesis, accelerated aging, and degenerative diseases that often correlate with persistent increases in 8-OxodG accumulations (Figure 5). Persistent oxidative stress is likely to synergize with increased DNA damage, an altered DNA damage response (DDR), and a diminished capacity for DNA repair following space radiation, so it is linked to a higher risk of cancer as well as degenerative diseases, relative to low-LET IR [3,20,46,49–51,56,120,121]. One of the primary concerns is the increased risk of carcinogenesis [50,120]. 8-OxodG can not only serve as a marker of oxidative DNA damage but also has direct links with an increased rate of mutagenesis that initiates and promotes the growth of cancerous cells [122]. Notably, 8-OxodG is known to form a base pair with adenine (A); therefore, it can induce a change from guanine (G) to thymine (T) during DNA replication, resulting in mutations (specifically, G > T, analogous to C > A mutations) known to drive tumorigenesis and cancer development [123–126]. In addition to genomic mutations, 8-OxodG has also been reported to cause transcriptional mutations through C > A transversion in mRNA [127–129]. AP sites formed during the repair of 8-OxodG lesions are also known to suppress gene expression by structural changes in the transcriptional elements [130], further suggesting the roles of 8-OxodG in the epigenetic regulation of gene expression [131].

Exposure to space radiation is known to cause accelerated aging phenotypes in various tissues, including the intestine, brain, and bone marrow [3,19,21,51]. Cellular senescence is a state of irreversible growth arrest linked to chronic stress-induced “accelerated aging phenotype” [132,133]. While this cell cycle arrest is traditionally seen as a defense against cancer, recent research suggests that a subset of senescent cells acquire SASP and secrete pro-inflammatory factors to drive aging-associated systemic inflammation [134]. Moreover, increased SASP signaling after high-LET radiation exposure has been implicated in higher risk of carcinogenesis [19,108], neuroinflammation [20], immunosuppression [135], and bone loss [51]. 8-OxoG formation exclusively in telomeres induces multiple hallmarks of premature senescence, including increased SA-β-gal activity, and SASP markers [136]. Interestingly, guanine present in telomeric repeats (5^′^-TTAGGG-3^′^) has also been reported to readily undergo oxidation, resulting in the growth arrest of normal human fibroblasts and epithelial cells [137–139]. The increased circulating leukocyte DNA content of 8-OxodG has been reported in IR-exposed human subjects [140,141]. Using the chemoptogenetic method, Banes et al. recently demonstrated that the induction of telomeric 8-OxodG results in the activation of DDR and p53 signaling, causing premature senescence [136]. Since persistent oxidative stress-induced increases in 8-OxodG are closely linked to both carcinogenic as well as premature senescence-associated degenerative diseases, monitoring the levels of 8-OxodG in easily accessible biofluids over time can provide insights into the oxidative stress status and potential health risks.

## 6. Analytical Methods for Quantitative Assessment of 8-OxodG

High-LET radiation is known to cause persistent oxidative stress, causing increased accumulations of 8-OxodG. It is crucial to quantitatively assess 8-OxodG in easily accessible biospecimens such as urine and blood (peripheral blood mononuclear cells (PBMCs), serum and plasma) [36,65,142,143]. As 8-OxodG can be produced from various endogenous sources, this makes it difficult to distinguish IR-induced increases in serum and urine levels, especially for localized high-LET exposure, i.e., through carbon ion radiotherapy. However, in scenarios of total-body high-LET radiation exposure, as in deep space, 8-OxodG monitoring using minimally invasive methods can be applied to assess potential health risks. Traditionally, 8-OxodG is often determined in DNA extracted from PBMCs [144,145]. However, in the case of IR-exposed subjects, such as radiotherapy patients at 24 h after the first dose, higher urinary 8-OxodG has been reported even when there is a stable level of 8-OxodG in leukocyte DNA [146]. Additionally, it has been reported that 8-OxodG is released out of cells into the bloodstream and finally through urine without further metabolism [147–149], and the urinary levels of 8-OxodG are not affected by the circadian rhythm [148,150,151]. Therefore, assessing urinary levels of 8-OxodG can better inform us about systemic oxidative stress-associated damage to DNA [143]. Contrary to 8-OxodG measurements in other biospecimens, urinary 8-OxodG levels can be easily normalized to the creatinine concentration [152], accounting for variations between individuals, leading to higher accuracy in its assessment. In 142 healthy human urine samples, the concentration of 8-OxodG was reported in the range of 0.24 to 2.47 nmol/mmol creatinine, with a mean concentration of 1.07 ± 0.49 nmol/mmol creatinine [143]. Recently, using a meta-analysis approach, Graille et al. assessed the background levels for urinary 8-OxodG in healthy populations and reported a pooled geometric mean value of 3.9 ng/mg creatinine (interquartile range 3 to 5.5 ng/mg creatinine) for adults with a mean body mass index (BMI) ≤ 25, and no sex specific effect was observed [153]. Among markers of oxidative stress, urinary 8-OxodG has been reported to display a high intraclass correlation > 0.95, reproducible measurements, and low coefficients of variation [154].

In the context of IR exposure and urinary levels of 8-OxodG, AbuArrah et al. performed a PRISMA (Preferred Reporting Items for Systematic Reviews and Meta-Analyses) guideline-based study and reported urinary 8-OxodG as a promising biomarker of oxidative stress after IR exposure [68]. In general, urinary 8-OxodG excretion begins to increase within hours after IR exposure and reaches a peak level within 1–2 days. Excretion then gradually declines over the following days and weeks, finally returning to a baseline. However, the time course of urinary 8-OxodG excretion is likely to vary depending on factors such as IR type, dose, the individual’s age, and renal function [68]. In particular, findings demonstrating higher levels of urinary 8-OxodG in airline pilots exposed to occupational low doses of cosmic radiation compared to un-exposed individuals [155] are highly significant in the context of space travel due to the similarities in the dose and dose rate of the IR exposure [156,157]. Consistent with chronic exposure to space radiation, signatures of persistent DNA damage responses including mitochondrial dysfunction and oxidative stress have also been reported in astronauts after missions to the International Space Station (ISS) [158]. In agreement, studies analyzing data from Mir, Skylab, and a longitudinal study on astronaut health, have demonstrated significantly higher levels of urinary 8-OxodG after long-duration space missions [159]. Notably, a 150% increase in urinary 8-OxodG has been reported after space flights to Russian space station Mir [160,161]. Urinary 8-OxodG excretion measured before, during, and after long-duration missions (4–9 months) on Russian space station MIR indicated increased oxidative damage to DNA. Urine samples from the first 5 days post flight demonstrated a significant increase from the mean pre-flight value [161].

Detecting 8-OxodG in urine requires sensitive and specific methods due to its low abundance and the complexity of biological samples. Some of the commonly used methods include high-performance liquid chromatography (HPLC), gas chromatography-mass spectrometry (GC-MS), and enzyme-linked immunosorbent assay (ELISA). HPLC coupled with electrochemical or ultraviolet detection is one of the most widely used methods for quantifying 8-OxodG. This technique separates 8-OxodG from other nucleosides based on their differential retention times and allows for precise quantification. Moreover, HPLC offers high sensitivity and selectivity, making it suitable for detecting low levels of 8-OxodG in biological matrices. However, its main drawbacks include the need for specialized equipment and expertise, as well as time-consuming sample preparation steps [35,162]. LC- and GC-MS enable the sensitive and selective detection of 8-OxodG by separating analytes based on their volatility and mass-to-charge ratio. This method offers excellent resolution and specificity, making it ideal for the quantitative analysis of 8-OxodG in complex biological samples. Additionally, GC-MS allows for the simultaneous detection of multiple oxidative DNA lesions, providing comprehensive insights into oxidative damage. Nonetheless, GC-MS requires the derivatization of 8-OxodG prior to analysis, which can introduce variability and complexity to the experimental procedure [163,164]. ELISA represents a cost-effective and high-throughput alternative for quantifying 8-OxodG in biological samples. This immunoassay relies on the specific binding of antibodies to 8-OxodG, followed by colorimetric or chemiluminescent detection. ELISA offers simplicity, rapidity, and scalability, making it suitable for screening large sample cohorts. However, its sensitivity and specificity may be compromised by cross-reactivity with structurally similar DNA lesions, necessitating validation with other analytical techniques [165]. In addition to these methods, new emerging technologies such as Nanopore sequencing (NPS), fragment length analysis with repair enzyme (FLARE)-coupled quantitative (q)-PCR, and immunosensor-based 8-OxodG quantification methods have been developed. NPS has the potential to directly detect 8-OxodG through its unique pore chemistry. As damaged DNA strands pass through the nanopore, the specific alterations in electronic signature caused by 8-OxodG can be identified [166]. 8-OxodG can also be identified and quantified through FLARE-coupled quantitative (q)-PCR. This assay can identify 8-OxodG within short stretches of nuclear and mitochondrial DNA in ng quantities, but so far, it has not been used to assay urine samples [167]. Additionally, immunosensors are being developed for 8-OxodG detection that utilize antibodies specific to 8-OxodG for selective detection. Various formats, including electrochemical and surface plasmon resonance (SPR) immunosensors, offer rapid and sensitive detection with potential for scalability. This simple and rapid colorimetric method has successfully been applied to inspect 8-OxodG concentration in urine samples and provided recoveries between 93.6 and 94.1%, with a limit of quantification of 34.3 nM, which is also comparable with the ELISA-based detection [168]. Future research and technological advancements in integrating multiple detection methods and combining the strengths of each technique while minimizing their limitations in the detection of 8-OxodG are likely to provide a testing platform with enhanced sensitivity and accuracy.

## 7. Conclusions and Future Perspectives

In conclusion, human exposure to high-LET radiation presents significant challenges and potential health risks across various scenarios, including space exploration, cancer radiotherapy, occupational exposures, and incidents involving radiological dispersal devices or nuclear disasters. The biological effects of high-LET radiation exposure are complex and not fully understood, necessitating comprehensive research efforts to elucidate its long-term impacts on human health. Future perspectives in this field revolve around the development and implementation of high-LET radiation biomarkers, particularly focusing on 8-OxodG as a reliable indicator of oxidative DNA damage. Such biomarkers hold promise for the early detection of potential health effects, enabling timely interventions to mitigate risks. Monitoring biomarkers could inform adjustments in mission durations, shielding strategies, or medical treatments for astronauts undertaking deep space missions. Moreover, advancing our understanding of high-LET radiation-induced persistent oxidative stress and its association with adverse health effects is crucial for developing targeted interventions and preventive measures. Analytical methods for the quantitative assessment of 8-OxodG in biological samples play a pivotal role in research and clinical settings. While various techniques exist, such as HPLC, LC/GC-MS, ELISA, and ultrahigh-performance liquid chromatography (UPLC)-MS/MS, selecting the most suitable method depends on factors like sensitivity, specificity, and potential for artifacts. Careful consideration of methodological limitations and validation is essential to ensure accurate and reproducible measurements of 8-OxodG levels. Overall, addressing the challenges posed by high-LET radiation exposure requires interdisciplinary collaborations, innovative research approaches, and continuous refinement of analytical techniques. By advancing our understanding of the biological effects of high-LET radiation and implementing effective biomonitoring strategies, we can better safeguard the health and safety of individuals exposed to such radiation, both in space exploration and terrestrial contexts.
